# WHAT MATTERS MOST FOR PERSON-CENTERED DEMENTIA CARE: PERSPECTIVES FROM STAKEHOLDERS IN LOW-RESOURCE LONG-TERM CARE

**DOI:** 10.1093/geroni/igae098.0570

**Published:** 2024-12-31

**Authors:** Jing Wang, Michael Lepore, Nancy Kusmaul, Sarah Holmes, Laura Davie, Alison Rataj, Yoon Chung Kim, Kirsten Corazzini

**Affiliations:** University of New Hampshire, Durham, New Hampshire, United States; University of Massachusetts Amherst, Amherst, Massachusetts, United States; University of Maryland Baltimore County, Baltimore, Maryland, United States; University of Maryland School of Nursing, Baltimore, Maryland, United States; University of New Hampshire, Durham, New Hampshire, United States; University of Massachusetts Boston, Durham, New Hampshire, United States; University of Maryland Baltimore, Baltimore, Maryland, United States; University of New Hampshire, Durham, New Hampshire, United States

## Abstract

One of the initial steps in supporting quality dementia care for residents living with dementia (RLWD) in low-resource long-term care (LTC) settings is to elucidate stakeholder perspectives on what matters most for providing person-centered dementia care in such settings. We conducted community-based, participatory research across four LTC facilities situated in federally designated medically underserved areas (two rural and two urban) to describe what matters most to key stakeholders in achieving person-centered dementia care in low-resource LTC settings through their own journeys. In-depth semi-structured interviews were conducted with administrative leaders (n=7), direct care staff (n=20), RLWD (n=16), and family/care partners (n=16), with transcripts thematically analyzed using NVivo14 software. Administrators and staff members acquaint themselves with dementia care principles, participate in daily huddles, and establish rapport with residents and families. As they progress, they acquire skills to observe residents’ behaviors, empathize with their experiences, cultivate trust, delivering intuitive care that honors individual preferences, routines, and past experiences. RLWD transitioning to LTC settings seek environments resembling private homes that foster self-advocacy and rely on staff members who understand their needs and promote a sense of belonging. For family members, the journey involves supporting their loved ones’ transition, advocating for their preferences, and maintaining open communication with staff. They play a crucial role in reinforcing resident preferences and ensuring the delivery of quality care. Throughout these journeys, stakeholders identify key quality measures, including odor, cleanliness, resident happiness, and the quality of relationships, as benchmarks for assessing person-centered care.

